# Thrombospondin-1 expression in urothelial carcinoma: prognostic significance and association with p53 alterations, tumour angiogenesis and extracellular matrix components

**DOI:** 10.1186/1471-2407-6-140

**Published:** 2006-05-29

**Authors:** E Ioachim, MC Michael, M Salmas, K Damala, E Tsanou, MM Michael, V Malamou-Mitsi, NE Stavropoulos

**Affiliations:** 1 Departments of Pathology and Cytology, University Hospital of Ioannina, Ioannina, Greece; 2 Department of Anatomy, University of Athens, Athens, Greece; 3 Red Cross Hospital (I.C.U.) Athens, Greece; 4 Department of Urology, 'G. Hatzikosta' General Hospital, Ioannina, Greece

## Abstract

**Background:**

Thrombospondin-1 (TSP-1) is an extracellular matrix component glycoprotein, which is known to be a potent inhibitor of angiogenesis and may be important in cancer invasiveness. We examined the TSP-1 expression in correlation with conventional clinicopathological parameters to clarify its prognostic significance in bladder cancer. In addition, the possible correlation of TSP-1 expression with microvessel count, VEGF expression, p53 expression as well as with the expression of the extracellular matrix components was studied to explore its implication in vascularization and tumour stroma remodeling.

**Methods:**

The immunohistochemical expression of TSP-1 in tumour cells and in the tumour stroma was studied in 148 formalin-fixed paraffin-embedded urothelial cell carcinoma tissue samples.

**Results:**

TSP-1 was detected in perivascular tissue, at the epithelial-stromal junction, in the stroma and in tumour cells in the majority of the cases. In tumour cells, low TSP-1 expression was observed in 43% of the cases, moderate and high in 7%, while 50% showed absence of TSP expression. A higher TSP-1 immunoreactivity in well and moderately differentiated tumours compared to poorly differentiated was noted. PT_1 _tumours showed decreased TSP-1 expression in comparison to pTa and pT_2–4 _tumours. Increased tumour cell TSP-1 expression was related to increased microvessel density. In the tumour stroma, 37% of the cases showed small amount of TSP-1 expression, 7.5% moderate and high, while 55% of the cases showed absence of TSP-1 stromal immunoreactivity. Stromal TSP-1 expression was inversely correlated with tumour stage and tumour size. This expression was also positively correlated with microvessel density, VEGF expression and extracellular matrix components tenascin and fibronectin. Using univariate and multivariate analysis we didn't find any significant correlation of TSP-1 expression in superficial tumours in both tumour cells and tumour stroma in terns of the risk of recurrence and disease progression

**Conclusion:**

Our data suggest that both tumour and stromal TSP-1 expression may play a role in tumour aggressiveness and angiogenesis. In addition, the correlation of stromal TSP-1 expression with extracellular matrix components fibronectin and tenascin indicate its possible implication in tumour stroma remodeling.

## Background

Thrombospondins (TSPs) are a family of extracellular proteins recognized as an endogenous constituent of the extracellular matrix (ECM) in many human tissues. They are adhesive proteins that promote cell-cell and cell-matrix interactions [1]. TSPs consists of five subtypes encoded by independent genes, in which TSP-1 and TSP-2 contain type-1 repeated with anti-angiogenic activity [2,3]. Five family members, each representing a separate gene product, probably exist in most vertebrate species. Each of these five proteins has a specific pattern of expression in embryonic and adult tissues with most tissues expressing at least one family member. Thrombospondin-1 the first family member to be identified, is a 450-kd adhesive glycoprotein that was initially discovered in platelets [4] and subsequently in a variety of cell types including endothelial cells, fibroblasts, smooth muscle cells, keratinocytes, macrophages and neutrophils [5]. TSP has implicated in the regulation of cell growth and proliferation [6], cell motility [7,8] cytosceletal organization [9], inflammation and wound healing [10], and the development and differentiation of cell types [11,12].

Research has demonstrated that both oncogene activation and loss of tumour-suppressor gene expression are directly or indirectly related with a decrease in TSP expression. This could increase the likelihood of the tumour gaining and the capacity to induce blood vessel formation, thereby making the 'angiogenic switch' and progressing towards greater malignancy [13,14].

Stromal components also play a critical and often underappreciated role in the formation of vascular stroma through the regulation of functions such as cell adhesion, migration, and gene expression by controlling the availability of growth factors [15].

In bladder cancer, it has been found that secretions from its cancer cells stimulate angiogenesis whereas those from normal urothelial cells are inhibitory [16]. In addition, it has been reported that, reduced thrombospondin-1 expression is associated with disease recurrence, decreases survival and disease progression [14,17].

The objective of the present study was to demonstrate immunohistochemically the patterns of thrombospondin-1 expression in bladder cancer tissues and to define its prognostic significance and to establish any relationship with standard clinicopathological features (grade, stage, concurrent in situ Ca, multifocality, recurrence rate, progression to muscle infiltrating disease). The alterations identified have been further correlated with tissue neovascularization (microvessel density-MVD), assessed by CD34, vascular endothelial growth factor (VEGF) expression, p53 expression as well as with the expression of the extracellular matrix (ECM) components tenascin (TN) and fibronectin (FN) in order to elucidate their interrelationships and the possible role of this protein in tumour stroma remodeling and angiogenesis.

## Methods

### Patients

Tumour specimens were obtained transurethrally from 148 patients (119 males and 29 females) aged from 27 to 89 years (mean 66.7 years) and were studied retrospectively. All the patients gave their consent and the investigation was approved by the Hospital Ethic committee. Biopsies near to and distant from the tumour were also studied. Bladder wall biopsies from patients undergoing transurethral prostatectomy were used as controls. The histopathological diagnosis was made on paraffin sections stained with haematoxylin and eosin. Histological grading and staging was performed in accordance with the guidelines of the International Union Against Cancer [18]. Seventy five patients presented with pTa disease, 57 patients with pT1 disease and 16 patients with pT2–T4 muscle infiltrating lesions. Seventy four patients from those with superficial tumours had single, 58 presented with multiple, 109 with primary while 29 with recurrent tumours. Tumours of well differentiated (grade 1) were detected in 6 patients, moderately differentiated (grade 2) in 78 patients, whereas 64 patients were found with grade 3 disease. Coexistence of carcinoma in situ, in the adjacent mucosa was found in 21 cases. From the 132 patients with superficial tumours 113 were followed up for maximum 69 months. Eighty patients from these subsequently went on to tumour recurrence whilst 33 did not. Tumour progression (defined as the presence of muscle invasion, stage pT2 or higher in a subsequent biopsy) was found in 8 cases. Patients with superficial bladder cancer were follow up and were part of another protocol studying [19] interferon gamma and categorized into two groups: one group that received only transurethral resection (TURT) (48 patients) and in the other resection was followed by intravesical instillations of interferon gamma (IFN) (65 patients). In 8 patients cystectomy was performed. The times to first recurrence and progression were recorded during the follow-up period (minimum 3, maximum 68, median 12 months to first recurrence and minimum 3, maximum 69, median 31 months to progression). Follow-up consisted of a biochemical profile, chest radiography, and a physical examination. Cystoscopy was conducted every three months for one year, every six months for 2 years and yearly thereafter.

### Immunohistochemistry

For immunohistochemical staining, additional 4 μm thick sections were cut from paraffin-embedded tissue placed on poly-L-lysine-coated glass slides. In brief, sections were deparaffinised in xylene and rehydrated. For the detection of thrombospondin-1, VEGF and p53, slides were immersed in citrate buffer (0.1 m, pH 0.6) in plastic Coplin jars and subjected to microwave irradiation twice for 15 min. Subsequently, all sections were treated for 30 min with 0.3% hydrogen peroxide in methanol to quench endogenous peroxidase activity and then incubated with primary antibodies. We used the method involving the avidin-biotin-peroxidase complex and developed the chromogen with immersion of the slides in a diaminobenzidine-H_2_O_2 _substrate for 5 min. The slides were counterstained in Harris' haematoxylin, dehydrated and mounted. To assess the specificity of the reaction, negative controls were included, where tumour sections subjected to the whole procedure except for incubation without the primary antibody. Positive control slides were also examined in each experiments. The antibodies sources and dilutions are shown in Table 1.

### Immunohistochemical assessment

#### Thrombospondin-1 and VEGF

To evaluate the expression of TSP-1 and VEGF proteins, we established a combined score, corresponding to the sum of both (a) staining intensity (0 = negative, 1 = weak, 2 = intermediate, 3 = strong, 4 = very strong staining) and (b) the staining extensive, percentage of positive cells (0 = 0%, 1 = 1–25%, 2 = 26–50%, 3 = 50–75%, 4 = >75%). The sum of both qualitative and quantitative immunostaining reached a maximum score of 8. The combined scores were then divided into 4 main groups: (-) = no immunostaining, score 0, (+) = weak immunostaining, scores 1–2, (++) = moderate immunostaining, scores 3–4, (+++) = strong immunostaining, scores 5–8.

#### Extracellular matrix components

the evaluation of TN and FN, the tumours were classified as 'positive' when there was unequivocal immunostaining of the matrix components in at least one representative area of the tumour. The positive tumours were semi-quantitatively scored as +, ++, and +++ corresponding to weak, moderate and extensive immunoreactivity respectively.

#### p53 protein

Nuclear staining for p53 was calculated as the percentage of positive epithelial cell in relation to the total number of neoplastic cells. Every stained cell was considered positive irrespective of intensity. Each sample was first scanned on low magnification and at least 5 representative fields were assessed with a high power magnification.

#### Microvessel count

The criteria that we used for microvessel recognition were the same as used in previous studies. Briefly, as microvessels we considered individual or clusters of cells with or without lumens, positively stained by anti-CD34. The lumen diameter had to be smaller than approximately eight red blood cells. Areas of fibrosis, necrosis and inflammation, and vessels with muscle wall were excluded from counting. In each tumour, the three areas with the highest vascularization ("hot spot") were selected. Individual microvessel counts were then made on a 250 X field (25 X objective and 10 X ocular, corresponding to an area of 0.363 mm^2^).

The immunoreactivity was interpreted by means of light-microscopic examination and evaluated independently by two pathologists (EI, MM). Differences in interpretation were reconciled by re-review of slides separately or jointly at a double-headed microscope. The staining was evaluated only in the areas with well-preserved tissue morphology and away from necrosis or artefacts. The immunostaining was assessed from numerically coded slides without any knowledge of survival or other clinical data.

### Statistics

Superior Performance Software System (SPSS) software 10.0 for windows (SPSS Inc., 1989–1999) was used by the authors to compare morphological features and protein expression data. Significant differences between the expression of the target proteins with regard to clinicopathological parameters were computed by the t-test for paired or non-paired values or ANOVA test if the data were normally distributed. Correlation between these proteins was computed using the Pearson's correlation coefficient for normally distributed data or the Kendall's Tau rank correlation coefficient where the data did not show a normal distribution. The prognostic significance of thrombospondin-1 expression, in determining the risk for recurrence, was studied with both univariate (log rank test) and multivariate (Cox proportional hazards) ways of analysis, separately for each group of patients. P-values ≤ 0.05 were considered statistically significant.

## Results

**Figure 1:**
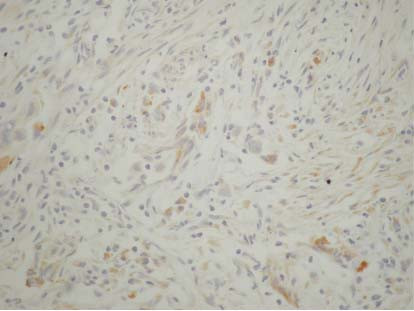
Tumour and stromal TSP-1 expression in a case of infiltrating urothelial bladder carcinoma (original magnification ABCX 200).

In urothelial cell carcinomas, TSP-1 was detected in the perivascular tissue, at the epithelial-stromal junction, in the stroma and in tumour cells (Fig. 1) in the majority of the cases

In tumour cells, low TSP-1 expression was observed in 64/148 (43.2%) of the cases, a moderate and high in 10/148 (6.8%), while 74/148 (50%) showed an absence of TSP-1 expression. Poorly differentiated tumours showed lower TSP-1 expression in tumour cells compared to the well and moderately differentiated ones (p = 0.038) (Table 2). PT_1 _tumours showed decreased TSP expression in comparison to pTa and PT_2–4 _(p = 0.005) and (p = 0.021) respectively (Table 2). A strong positive relationship of tumour cell TSP-1 expression with MVD (p < 0.0001) was also observed (Table 3).

In tumour stroma, 55/147 (37.4%) of the cases showed small amount of TSP-1 expression, 11/147 (7.5%) moderate and high, while 81/147 (55.1%) of the cases showed an absence of TSP-1 stromal immunoreactivity. PT2–4 tumours showed higher stromal TSP-1 expression compared to PTa (p = 0.033) and PT_1 _(p = 0.013) (Table 2). In addition, larger tumours (>3 cm) showed higher stromal TSP-1 expression (p = 0.021) (Table 2). This expression was also positively correlated with MVD (p = 0.031) and VEGF expression (p = 0.001) as well as with both extracellular matrix components tenascin (p < 0.0001) and fibronectin. (p = 0.003) (Table 3). A positive relationship between expression of TSP-1 in tumour cells and stroma was observed (p = 0.038) for each case.

Tumour and stromal TSP-1 expression did not correlated with p53 protein expression (Table 3). In addition, tumour and stromal TSP-1 expression did not correlate with the concurrent in situ element, multifocality, primary or recurrent status. The prognostic significance of TSP-1 expression in superficial tumours in both tumour cells and tumour stroma in determining the risk of recurrence and progression with univariate and multivariate methods of analysis showed no statistically significant differences. In addition, this expression did not correlate with progression to muscle infiltrating disease.

## Discussion

Angiogenesis is a multistep process, which involves changes in the extracellular matrix, and endothelial cell proliferation, migration, and differentiation into capillaries. Studies of tumour biology reveal a complex network of autocrine and paracrine interactions between tumour cells, stromal cells, and endothelial cells, which are in turn influenced by the composition of the extracellular matrix. The development of new blood vessels within a tumour depends upon the local balance between angiogenic and anti-angiogenic factors. The induction of angiogenesis has an important role in the pathogenesis of bladder cancer [16]. Microvessel density has been correlated strongly with advanced pathological findings and poor outcomes in patients with bladder cancer [20,21] and drugs with anti-angiogenic activity have blocked by bladder tumour progression in the experimental models [22,23]. Down-regulation of the secretion of TSP-1 was found to be a key event in the change from an anti-angiogenic to an angiogenic phenotype during bladder tumorigenesis [16]. The results of the present study showed a relationship of tumour cells TSP-1 expression with tumour grade, according to the findings of other studies in bladder cancer and in other types of tumours [14,24,25]. Similarly, an increase TSP-1 expression, by tumour cells has been found to be correlated with a reduced metastatic potential in experimental tumours, formed from melanoma, lung, and breast carcinoma cell lines [26]. In addition, we also found an inverse relationship of stromal TSP-1 with tumour stage. It is possible that this might indicate the switch to an angiogenic phenotype in invasive tumours and support the association between stromal TSP-1 loss and a more aggressive phenotype. In contrast, pT1 tumours showed a lower TSP-1 expression in tumour cells compared to pTa and pT2–4. This phenomenon possibly reflect different stage of TSP-1 production by tumour or stromal cell according to various environmental or intrinsic factors.

A relevant immunohistochemical study reported by Grossfeld et al [14] showed that patients with low TSP-1 expression exhibited increased recurrence rates and decreased overall survival. In this study it was found that TSP-1 was an independent predictor of disease recurrence and overall survival after stratifying for tumour stage, lymph node status, and histological grade, but it was not independent of the p53 status. In addition, Goddard et al [17] observed that reduced perivascular TSP-1 staining at presentation was an independent predictive factor for the subsequent development of muscle invasive or metastatic disease. In the current study, we found no association of TSP-1 expression and recurrence rate or in the progression of muscle invasive tumour. These discrepancies are possible due to the different antibodies used, or to difficulties in quantification within a friable papillary tumour or differences in the behavior of tumour tissue microenvironment from case to case. In the study, of Grossfeld et al [14] the antibody used was the mouse anti-TSP monoclonal antibody MA-II that recognizes an epitope in the amino-terminal region of TSP-1, while in the study of Goddard JC et al [17] used monoclonal antibody against TSP-1(Ab-4). In the present study we used a monoclonal mouse antibody to thrombospondin clone A6.1. Grossfeld et al [14] also found that decreased levels of TSP-1 staining were correlated with increased MVD and increasing p53 mutation. We found a positive correlation of tumour and stromal TSP-1 expression with MVD and a strong relationship of stromal TSP-1 expression with tumour VEGF expression. Although TSP-1 has an anti-angiogenic activity which is involved in the interaction of the microvascular endothelial cell receptor CD36 [27], in the present study we found TSP-1 as inducer of microvascularity. The role played by TSP-1 in the regulation of angiogenesis is not clear. Positive and negative effects of TSP-1 on angiogenesis have been reported. It has previously been shown that this protein may be inhibitory to angiogenesis both in vivo and vitro studies [2,28,29]. In contrast to these findings, a recent study by Nicosia and Tusznyski [30], has suggest that matrix-bound thrombospondin-1 may indirectly promoting angiogenesis in vitro. In addition, Ben Ezra et al [31] has shown that TSP-1 enhances the induction of angiogenesis by either bFGF or bacterial endotoxin lipopolysaccharide using the rabbit cornea assay.

It is now well established that the activation of oncogenes or inactivation of tumour suppressor genes may directly influence the angiogenic phenotype by altering the relative amounts of inducers and inhibitors secreted by the tumour, in addition to their effect on proliferation and apoptosis [32]. It has been reported that there is a link between TSP-1 expression, tumour angiogenesis, and the p53 tumour suppressor gene status and it has been demonstrated that in fibroblasts, that wild type p53 inhibits angiogenesis through the up-regulation of TSP synthesis [33]. Volpert et al [34] showed that mutation or inactivation of p53 in fibrosarcoma leads to the down-regulation of TSP-1 and enhanced expression of VEGF, tipping the balance in favor of angiogenesis. Environmental factors such as relative hypoxia, within the microenvironment of most tumors, including bladder cancer, may also influence the expression of angiogenic mediators, most notably VEGF[35]. It has also been hypothesized that hypoxia may regulate TSP-1, VEGF and angiogenic activity in bladder cancer [36]. In the present study we found no relationship between TSP-1 and p53 protein expression. Our results could suggest that p53 does not act as primary regulator of angiogenesis in bladder cancer. Such a clear-cut association might not be a general feature for all tumour types. Analysis of certain cancers, such as lung cancer, cholangiocarcinoma, and glioblastoma, found no association between the presence of p53 alterations and TSP-1 expression [37-40] in line with our results.

The composition of the extracellular matrix may be modified in vivo in several ways. For example, angiogenic factors are known to modulate both the synthesis of matrix proteins [41,42]. As well as the synthesis of certain proteases and their inhibitors [42-44]. To our knowledge there are no reports concerning the correlation between TSP-1 expression and the extracellular matrix in bladder cancer. Fibronectins may play an important role in vascular stroma formation because they have been reported to be an essential for the heart and blood vessel morphogenesis [45], to be chemotactic for endothelial cells [46], to modulate endothelial response to growth factors [47], and to promote the elongation of microvessels during angiogenesis in vitro [48]. TN and FN are glycoprotein components of the ECM which seem to have competitive functions. It can be speculated that this competitive relationship between these molecules is important for cellular functions [49]. There are evidences that tenascin and TSP-1 modulate sprouting of endothelial cells [50]. Tenascin expression indicates an altered cell-ECM interaction that might facilitate epithelial tumour cell invasion [51,52]. Tenascin expression is highly up-regulated during tumour invasion in many tumour types, including bladder carcinomas [53,54]. In a previous study concerning the expression of extracellular matrix components in urothelial carcinoma [55] we found that tenascin and fibronectin seemed to be correlated with more aggressive tumour behaviour. In another study [56], we found that extracellular matrix components may be involved in the development and progression of breast cancer. In addition, overexpression of stromal TN and FN seem to have a poor prognostic value in breast cancer patients. In the present study we found a strong relationship of TSP-1 expression with TN and FN suggesting the contribution of these glycoproteins in tumour stroma, remodeling or the effect of these ECM products in bladder vascularization. The formation of a new vessels is required for tumour progression and invasion and inhibition of angiogenesis may affect the early steps of tumour development. So, our data provide implication of other pathways of the regulation of TSP-1 and angiogenesis, possibly through the interaction of ECM components. The ultimate aim is to develop another modality in targeting anti-angiogenic therapies.

## Conclusion

The results of the present study indicate that TSP-1 expression is not useful for predicting recurrence or progression to muscle invasive diseases. However, reduction of its expression appears to be correlated with a more aggressive phenotypes. In addition, stromal TSP-1 expression seems to play a role in bladder cancer vascularization possibly in cooperation with other angiogenic factors. Finally, TSP-1 expression could contribute in bladder cancer tissue remodeling, possibly in collaboration with other ECM components.

## Abbreviations

Vascular endothelial growth factor: VEGF

Platelety-derived growth factor :PDGF

Thrombospondin -1: TSP

Microvessl density : MVD

Extracelular matrix : ECM

Tenascin : TN

Fibronectin : FN

Transurethral resection of bladder tumours : TURT

No significant : NS

## Competing interests

The author(s) declare that they have no competing interests.

## Authors' contributions

All Authors have participated sufficiently in the work to take public responsibility for appropriate portions of the content as following:

EI participated in the sequence alignment and drafted the manuscript. In addition, she was interpreted the immunohistochemical findings by means of light-microscopic examination.

MC M also carried out the immunohistochemical evaluation independently.

MS participated in the design of the study and performed the statistically analysis.

KD and ET conceived of the study and participated in the collection of the slides for the immunohistochemical technical method and for the clinicopathological parameters of each case. MM M and NE S were contributed at the clinical information and follow up data

V MM participated in the design and coordination of the study

In addition, all authors read and approved the final manuscript.

## Pre-publication history

The pre-publication history for this paper can be accessed here:



## Figures and Tables

**Table 1:** Antibodies used

Antibodies	Supplier	Dilution	Incubation time
TSP-1(A6.1)*	DBS	1:30	30 min
VEGF*	Dako	1:50	1 hour
CD34(QBEnd/10)	Novo Castra	1:50	1 hour
Tenascin (TN2)#	Dako	1:50	1 hour
Fibronectin (clone, 568)#	Novo Castra	1:100	1 hour
P53 (DO-7)*	Dako	1:50	1 hour

*with microwave oven antigen retrieval

# incubation with pronase

**Table 2:** Tumour and stromal TSP expression in correlation with clinicopathological data

	*tumour cells TSP*			*stromal TSP*		
	*0,1/2,3*		*p value*	*0*/+,++,+++		*p value*
*grade*						
1	6	1		2	5	
2	71	6	p = 0.038	38	37	NS
3	58	6		39	26	
*Stage*						
pTa	69	5		39	35	
pT1	53	3	p = 0.005	34	21	p = 0.033
pT2–4	12	6	p = 0.021	6	12	P = 0.013
*size*						
<3 cm	71	8	NS	45	33	p = 0.021
>3 cm	36	2		15	28	
*Single*	67	7		38	36	
*multiple*	62	3	NS	36	28	NS
*no in situ*	110	10		63	56	
*in situ*	21	-	NS	12	9	NS

**Table 3:** Tumour and stromal TSP expression in correlation with MVD, VEGF expression and ECM components

	*tumour cells TSP*			*stromal TSP*		
	*0,1/2,3*		*p value*	*0*/+,++, +++		*p value*
*mvd*						
low	57	3	p < 0.000	36	24	p = 0.031
high	55	5	1	23	37	
*VEGF*						
low(0,1)	30	2	NS	24	8	p = 0.001
high (2,3)	100	8		51	56	
*p53*						
<5%	40	5	NS	37	17	NS
>5%	44	4		29	19	
*tenascin*						
*0*,+	70	6	NS	56	19	p < 0.000
++,+++	23	3		9	17	1
*fibronectin*						
*0*,+	39	3	NS	32	10	p = 0.003
++,+++	54	6		33	26	
